# Clinicopathologic characterization and abnormal autophagy of *CSF1R*-related leukoencephalopathy

**DOI:** 10.1186/s40035-019-0171-y

**Published:** 2019-12-02

**Authors:** Wo-Tu Tian, Fei-Xia Zhan, Qing Liu, Xing-Hua Luan, Chao Zhang, Liang Shang, Ben-Yan Zhang, Si-Jian Pan, Fei Miao, Jiong Hu, Ping Zhong, Shi-Hua Liu, Ze-Yu Zhu, Hai-Yan Zhou, Suya Sun, Xiao-Li Liu, Xiao-Jun Huang, Jing-Wen Jiang, Jian-Fang Ma, Ying Wang, Shu-Fen Chen, Hui-Dong Tang, Sheng-Di Chen, Li Cao

**Affiliations:** 1 Department of Neurology, Rui Jin Hospital & Rui Jin Hospital North, Shanghai Jiao Tong University School of Medicine, Shanghai, 200025 China; 2 Department of Neurology, Peking Union Medical College Hospital, Chinese Academy of Medical Sciences & Peking Union Medical College (CAMS & PUMC), Beijing, 100032 China; 3 Anhui University of Science and Technology School of Medicine, Huainan, 232001 Anhui Province China; 4 Department of Pathology, Rui Jin Hospital, Shanghai Jiao Tong University School of Medicine, Shanghai, 200025 China; 5 Department of Neurosurgery, Rui Jin Hospital, Shanghai Jiao Tong University School of Medicine, Shanghai, 200025 China; 6 Department of Radiology, Rui Jin Hospital, Shanghai Jiao Tong University School of Medicine, Shanghai, 200025 China; 7 Department of Hematology, Rui Jin Hospital, Shanghai Jiao Tong University School of Medicine, Shanghai, 200025 China; 8 Suzhou Municipal Hospital, Suzhou, 234000 Anhui Province China; 9 Department of Neurology, Shanghai Fengxian District Central Hospital, Shanghai Jiao Tong University Affiliated Sixth People’s Hospital South Campus, Shanghai, 201406 China

**Keywords:** Hereditary diffuse leukoencephalopathy with spheroids, *CSF1R*, Autophagy, Haploinsufficiency

## Abstract

**Background:**

*CSF1R*-related leukoencephalopathy, also known as hereditary diffuse leukoencephalopathy with spheroids (HDLS), is a rare white-matter encephalopathy characterized by motor and neuropsychiatric symptoms due to colony-stimulating factor 1 receptor (*CSF1R*) gene mutation. Few of *CSF1R* mutations have been functionally testified and the pathogenesis remains unknown.

**Methods:**

In order to investigate clinical and pathological characteristics of patients with *CSF1R*-related leukoencephalopathy and explore the potential impact of *CSF1R* mutations, we analyzed clinical manifestations of 15 patients from 10 unrelated families and performed brain biopsy in 2 cases. Next generation sequencing was conducted for 10 probands to confirm the diagnosis. Sanger sequencing, segregation analysis and phenotypic reevaluation were utilized to substantiate findings. Functional examination of identified mutations was further explored.

**Results:**

Clinical and neuroimaging characteristics were summarized. The average age at onset was 35.9 ± 6.4 years (range 24–46 years old). Younger age of onset was observed in female than male (34.2 vs. 39.2 years). The most common initial symptoms were speech dysfunction, cognitive decline and parkinsonian symptoms. One patient also had marked peripheral neuropathy. Brain biopsy of two cases showed typical pathological changes, including myelin loss, axonal spheroids, phosphorylated neurofilament and activated macrophages. Electron microscopy disclosed increased mitochondrial vacuolation and disorganized neurofilaments in ballooned axons. A total of 7 pathogenic variants (4 novel, 3 documented) were identified with autophosphorylation deficiency, among which c.2342C > T remained partial function of autophosphorylation. Western blotting disclosed the significantly lower level of c.2026C > T (p.R676*) than wild type. The level of microtubule associated protein 1 light chain 3-II (LC3-II), a classical marker of autophagy, was significantly lower in mutants expressed cells than wild type group by western blotting and immunofluorescence staining.

**Conclusions:**

Our findings support the loss-of-function and haploinsufficiency hypothesis in pathogenesis. Autophagy abnormality may play a role in the disease. Repairing or promoting the phosphorylation level of mutant CSF1R may shed light on therapeutic targets in the future. However, whether peripheral polyneuropathy potentially belongs to *CSF1R*-related spectrum deserves further study with longer follow-up and more patients enrolled.

**Trial registration:**

ChiCTR, ChiCTR1800015295. Registered 21 March 2018.

**Electronic supplementary material:**

The online version of this article (10.1186/s40035-019-0171-y) contains supplementary material, which is available to authorized users.

## Background

*CSF1R*-related leukoencephalopathy, also known as hereditary diffuse leukoencephalopathy with spheroids (HDLS), is a rapidly progressive neurodegenerative disease clinically characterized by personality and behavioral changes, motor symptoms, cognitive decline, mental disorder, seizures and other phenotypes [1, 2]. Pathological hallmarks of *CSF1R*-related leukoencephalopathy, also described as pigmented orthochromatic leukodystrophy (POLD), portray giant axonal swellings (spheroids), pigmented macrophages, loss of axons and myelin sheaths within the cerebral white matter [1, 3–5]. The neuropathological features include degenerative changes of the cerebral white matter, bilateral and asymmetric frontal prominence and atrophy of corpus callosum, with U-fibers spared [2, 6–8]. In 2004, the term adult-onset leukodystrophy with neuroaxonal spheroids and pigmented glia (ALSP) was further proposed as a comprehensive pathologic term [4]. In 2011, colony-stimulating factor 1 receptor gene (*CSF1R*) was confirmed as the causative gene of HDLS [2]. Although other genes have been considered to cause severe progressive leukoencephalopathy, such as alanyl-tRNA synthetase 2 (*AARS2*), *CSF1R*-related cases accounts for the majority of ALSP [9–11]. In the central nervous system (CNS), CSF1R protein is almost exclusive to microglia, though low expression in some neurons in the hippocampus has been reported [12]. While CSF1R signal transduction has been studied extensively in macrophages, the pathologic mechanism whereby microglia expressing mutant CSF1R affect cell function and brain homeostasis remains elusive [13, 14]. Several compelling studies have suggested that cells expressing pathogenic mutation are defective in CSF1R autophosphorylation [2, 15, 16]. Since CSF1R assembles into homodimers in vivo, the controversy still hangs over whether haploinsufficiency or dominant negative effect leads to the pathological change [2, 17]. Thus far, no treatment has been proved to be beneficial for the disease, except that only 1 patient treated with hematopoietic stem cell transplantation was reported with no more progression for at least 15 years [18].

In the present study, we identified 15 patients with leukoencephalopathy due to different types of *CSF1R* mutations. Based on thorough clinical, neuroimaging, neuropathological and genetic analysis, we attempted to investigate the functional impact of *CSF1R* mutations.

## Methods

### Participants

A total of 15 patients from 10 families were enrolled in this study. All patients were evaluated and counseled by senior neurologists and clinical geneticists. For each family, only one patient (proband) was included for exome sequencing. Diagnosis of *CSF1R*-related leukoencephalopathy was stringently made according to Konno’s criteria [19].

### Standard protocol approvals, registrations, and patient consents

The ethics committee of Rui Jin Hospital, Shanghai Jiao Tong University School of Medicine, Shanghai, China approved the study (2017–211). This study is registered at http://www.chictr.org.cn (registration number: ChiCTR1800015295). All participants or their legal guardians provided written informed consent.

### Exome sequencing and data analysis

Genomic DNA was extracted using a standard phenol/chloroform extraction protocol. Exome sequencing was performed on 10 probands, using Agilent SureSelect v6 reagents for capturing exons and Illumina HiSeq X Ten platform for sequencing. Alignment to human genome assembly hg19 (GRCh37) was carried out followed by recalibration and variant calling. Firstly, population allele frequencies compiled from public databases of normal human variation [dbSNP (National Center for Biotechnology Information Database of Single Nucleotide Polymorphisms 142), ESP6500 (National Heart, Lung and Blood Institute Exome Sequencing Project 6500), and 1000 g (1000 Genomes Project)] were used to initially filter the data to exclude all variants present in the population at greater than 5% frequency. Next, the sequence variants were further interpreted and classified according to the American College of Medical Genetics and Genomics (ACMG) Standards and Guidelines [20]. In this segment, allele frequency (from: 1000 g, ESP6500, dbSNP, ExAC and 600 in-house ethnically matched healthy controls), nucleotide and amino acid conservation, pathogenicity prediction [PolyPhen-2 (http://genetics.bwh.harvard.edu/pph2), SIFT (http://sift.jcvi.org), Mutationtaster (http://www.mutationtaster.org), and inheritance pattern were all analyzed by two neurogeneticists and all data sets were annotated for previously reported disease-causing variants using the Professional Version of Human Gene Mutation Database. Putative pathogenic variants were further confirmed by Sanger sequencing both the forward and reverse strands, as well as co-segregation analysis among family members.

### Neuropathology

We performed neuropathologic examinations on biopsied specimens taken from the white matter of anterior horn of lateral ventricle of Patient 5 and 7. Paraffin-embedded fixed tissue sections (7 μm) were stained with hematoxylin & eosin (H&E), and with periodic Schiff. Sections were also immunostained with a DAKO Autostainer system (DAKO, Carpinteria, CA) using EnVision FLEX DAB^+^ Chromogen with the following antibodies: anti-phosphorylated neurofilament (mouse monoclonal [2836], 1:100; Cell Signaling Technology); anti-CD68 (PGM1) (mouse, 1:100; Dako); anti-CD3 (D7A6E) (rabbit [85061], 1:200; Cell Signaling Technology); anti-CD20 (EP459Y) (rabbit [ab78237], 1:200; abcam); anti-Olig2 (EPR2673) (rabbit [ab109186], 1:100; abcam).

### Cell culture transfection and Western blotting

HEK 293 T cells were maintained in Dulbecco’s Modified Eagle Medium (DMEM) with 10% fetal bovine serum (FBS) and 1% penicillin/streptomycin (PS) at 37 °C in a humidified incubator with 5% CO_2_. One day before transfection, cells were plated at 150,000 cells per well in 6-well culture dish. The next day, cells were transfected with 2.5 μg of EGFP control plasmid DNA or CSF1R-EGFP wild-type (CSF1R-WT) or mutant (CSF1R-Mut: c.1907 T > A, c.2026C > T, c.2342C > A, c.2342C > T, c.2381 T > C, c.2468C > A and c.2552 T > C) plasmid DNA using Lipofectamine 3000 transfection reagent (Invitrogen). We also set one benign variation (c.1606C > G) as non-pathogenic control, which was identified in our healthy control data base. Two days after transfection, media was changed to serum-free media for 16 h before treatment with 50 ng/mL human recombinant MCSF (R&D Systems). CSF-1 remained in the media for 5, 15, or 30 min before the cells were split in radioactive immunoprecipitation assay (RIPA) buffer (Beyotime) to extract protein for western blot analysis. HEK 293 T cells without CSF-1 treatment were collected as controls. Cell lysates were diluted in an equivalent volume of 6X SDS-PAGE Sample Loading Buffer (Beyotime) for protein denaturation. For cell lysates, equal volumes were run on 13% SDS polyacrylamide gels. Total CSF1R levels were detected using the Anti-GFP antibody (AVES, 1:2500). CSF1R autophosphorylation was detected using Phospho-M-CSF Receptor primary antibodies against p-Y546 (1:1000), p-Y699 (1:1000) and p-Y809 (1:1000) from Cell Signaling Technology. GAPDH primary antibody (rabbit, 1:1000, Cell Signaling Technology) was used to ensure equal protein loading. Blots were then incubated with anti-chicken and anti-rabbit secondary HRP-conjugated antibodies (1:5000, Beyotime) and bands were detected by enhanced chemiluminescence using Western Blot Enhancer reagents (Thermo Scientific). Construct Mito- pDsRed2 was donated by Dr. XD Liu. Microtubule associated protein 1 light chain 3 (LC3), the marker of autophagy, was detected by anti-LC3B (D11) (rabbit [3868], 1:1000 for western blotting, 1:200 for immunofluorescence, Cell Signaling Technology).

### Immunofluorescence

Cells transfected with the respective expression constructs were washed in PBS and fixed by using 4% paraformaldehyde for immunofluorescence test. Cells were blocked with 10% normal donkey serum and 0.3% Triton X-100 in PBS for 60 min, incubated with primary antibody in blocking solution at 4 °C overnight and incubated with Alexa Fluor 488 or 594 secondary antibodies (1:1000, Life). DAPI (4′,6-diami-dino-2-phenylindole) (1:10000, Life) was used for nucleic acid staining. Images were taken with a Zeiss 710 confocal microscope. All statistical analyses of fluorescence were conducted using Image J (National Institutes of Health).

## Results

### Clinical findings

A total of 15 patients (male: female = 5: 10) were enrolled including 10 probands, among which one half were familial and the other were sporadic. Detailed clinical presentations of these patients were summarized in Table 1. The average age at onset was 35.9 ± 6.4 years (range 24–46 years old). Of note, younger age of onset was observed in women than men (34.2 vs. 39.2 years). The average course of illness was 2.8 ± 1.7 years (range 1–8 years). During this study, two patients (Patient 8 and 12) died because of complications of being trapped in bed in advanced stage (4 years after onset). For the initial symptoms, 5 patients had speech dysfunction, cognitive decline or social withdraw. 6 patients manifested early signs of motor symptoms such as resting tremor, bradykinesia, uni/bilateral rigidity, postural instability and gait disturbance, which were not responsive to levodopa. The other 4 patients initially presented with both motor symptoms and neuropsychiatric disorders. Personality or behavior changes, dementia, parkinsonian symptoms and dysphasia were the most frequent clinical manifestations (13/15 for each item). Mood disorders, such as depression and anxiety, were reported in 9 out of 15 patients. Seizure was observed in approximately 20% of our cases, mainly presenting with partial episodes.

**Table 1:** Clinical and genetic characteristics of individuals from families with HDLS

Family ID		F1-T4153	F2-T4033		F3-R1092	F4-T3538	F5-T2980	F6-T3893		F7-T4244	F8-T4265			F9-T4137	F10-T3975		Total
Individual		P1(II:2)	P2(II:3)	P3(II:2)	P4(II:3)	P5(II:1)	P6(II:1)	P7(III:1)	P8(II:4)	P9(II:1)	P10(II:4)	P11(II:1)	P12(II:2)	P13(II:3)	P14(III:3)	P15(III:1)	15
Gender		M	F	F	M	F	F	F	F	M	M	F	F	M	F	F	M: 5, F:10
Onset/duration		29/2	30/2	30/2	46/4	36/2	42/2	35/2	38/4(Die)	37/1	39/1	38/3	42/4(Die)	45/2	24/3	27/8	35.9 ± 6.4/2.8 ± 1.7
Initial symptoms		Left limb rigidity, gait dysfunction	Cognitive decline, dysarthria	Cognitive decline, speech & executive dysfunction	Gait dysfunction	Right limb weakness, rigidity	Cognitive decline, gait dysfunction	Dysarthria, gait dysfunction	Dysarthria	Cognitive decline, gait dysfunction	Memory loss	Memory loss, slow response	Indifference, bradykinesia, slow response	Left limb rigidity, weakness, gait dysfunction	Gait dysfunction	Gait dysfunction	/
Clinical features during course of the illness	Personality and behavior changes	(+) reticence, forced laughter or crying	(+)	(+)	(+) inflexible, repeat utterance	(−)	(+)	(+)	(+)	(+)	(−)	(+)	(+) reticence	(+) forced laughter or crying, social withdrawal	(+) reticence, forced laughter or crying	(+) reticence, forced laughter or crying, social withdraw	13/15
	Dementia	(+)	(+)	(+)	(+)	(−)	(+)	(+)	(+)	(+)	(+)	(+)	(+)	(−)	(+)	(+)	13/15
	Depression/anxiety	(+)	(+)	(+)	(+)	(−)	(+)	(−)	(+)	(+)	(−)	(−)	(−)	(+)	(+)	(−)	9/15
	Parkinsonism	(+)	(+)	(+)	(+)	(+) tremor, drag-to gait, rigidity, small handwriting	(+) bradykinesia, walking difficulty, instability	(+) walking difficulty, rigidity, instability	(+)	(−)	(−)	(+) mask-like face, bradykinesia	(+) mask-like face, bradykinesia	(+) rigidity, gait dysfunction, bradykinesia, tremor	(+) right gait dysfunction, rigidity, instability	(+) standing/walking difficulty, weakness, instability	13/15
	Seizure	(+)	(−)	(−)	(−)	(−)	(−)	(−)	(−)	(−)	(−)	(−)	(−)	(−)	(+)	(+)	3/15
	Dysphasia	(+)	(+)	(+)	(+)	(−)	(+)	(+)	(+)	(+)	(−)	(+)	(+)	(+)	(+)	(+)	13/15
MMSE/MoCA		NA	14/11	NA	25/22	17/NA	15/NA	15/19	NA	NA	28/25	NA	NA	23/15	NA	19/NA	8/8
Neuroimage	White matter lesions	(+)	(+)	(+)	(+)	(+)	(+)	(+)	NA	(+)	(+)	(+)	(+)	(+)	(+)	(+)	14/14
	Corpus callosum	(−)	(+)	(+)	(−)	(−)	(+)	(+)	NA	(+)	(−)	(−)	(−)	(+)	(+)	(+)	8/14
	Cerebellum	(−)	(−)	(−)	(−)	(−)	(−)	(−)	NA	(−)	(−)	(−)	(−)	(−)	(−)	(−)	0/14
	Atrophy	(+)	(+)	(+)	(+)	(+)	(−)	(+)	NA	(+)	(−)	(+)	(+)	(+)	(+)	(+)	12/14
	Calcification	(−)	(−)	(−)	(−)	(−)	(−)	(−)	NA	(−)	(−)	(−)	(−)	(−)	(−)	(+)	1/14
EMG/EEG		NA	NCV(−)	NA	NA	NCV(+)	NA	NA	NA	NA	NA	NA	NA	NCV(−)	NCV(−), EEG(+)	NCV(−), EEG(+)	NCV(1/5), EEG(2/2)
*CSF1R* mutation		c.1907 T > A (p.I636N)	c.2026C > T (p.R676*)	c.2026C > T (p.R676*)	c.2342C > A (p.A781E)	c.2342C > T (p.A781V)	c.2381 T > C (p.I794T)	c.2381 T > C (p.I794T)	c.2381 T > C (p.I794T)	c.2381 T > C (p.I794T)	c.2381 T > C (p.I794T)	c.2381 T > C (p.I794T)	c.2381 T > C (p.I794T)	c.2468C > A (p.A823D)	c.2552 T > C (p.L851P)	c.2552 T > C (p.L851P)	/

*HDLS* Hereditary diffuse leukoencephalopathy with spheroids, *CSF1R* Colony-stimulating factor 1 receptor (NM_ 005211.3)

*F1* Family 1, *P1* Patient 1, *M* Male, F Female, *EMG* Electromyogram, *EEG* Electroencephalogram, *NCV* Nerve conduction velocity, *MRI* Magnetic resonance imaging, (−) Normal, (+) Abnormal, *NA* Not available

All the patients undergoing neuroimaging examinations revealed white matter lesions (14/14), mainly involving the frontal and parietal region (Additional file 1: Figure S1A-C). Eight out of fourteen patients had atrophy or abnormal high signal on corpus callosum on diffusion weighted imaging (DWI). Atrophy in parietal and frontal lobes was observed in 12 out of 14 patients, leading to ventricular enlargement and widening of cerebral sulci and fissures. In Family 10, Patient 15 also had intracranial calcification (Additional file 1: Figure S1C-b) and Patient 14 undergoing lateral ventricle drainage gained no obvious benefit (Additional file 1: Figure S1C-c).

Among the five patients undergoing electrophysiological examinations, Patient 5 was recorded with obvious peripheral nerve lesion. As shown in Additional file 2: Table S1, the electromyography (EMG) demonstrated slowed motor and sensory nerve conduction velocities in limbs (MCV: 34.9–36.4 m/s, SCV: 37.2–37.5 m/s), distal motor and sensory latency delay, decreased compound motor action potential (CMAP) and amplitudes of sensory nerve action potential (SNAP).

### Pathological findings

Patient 5 and 7 initially presenting with motor symptoms were performed with brain biopsy of white matter, showing axonal spheroids, remarkably decreased density of myelinated and non-myelinated fibers, myelin loss and phosphorylated neurofilament (Additional file 1: Figure S1D-O). Under electron microscopy, ballooned and demyelinated fibers in frontal white matter were captured with increased mitochondrial vacuolation (red arrows) and vesicles (Additional file 1: Figure S1P) and abundant disorganized neurofilaments (Additional file 1: Figure S1Q-R).

### Genetic findings

**Fig. 1:**
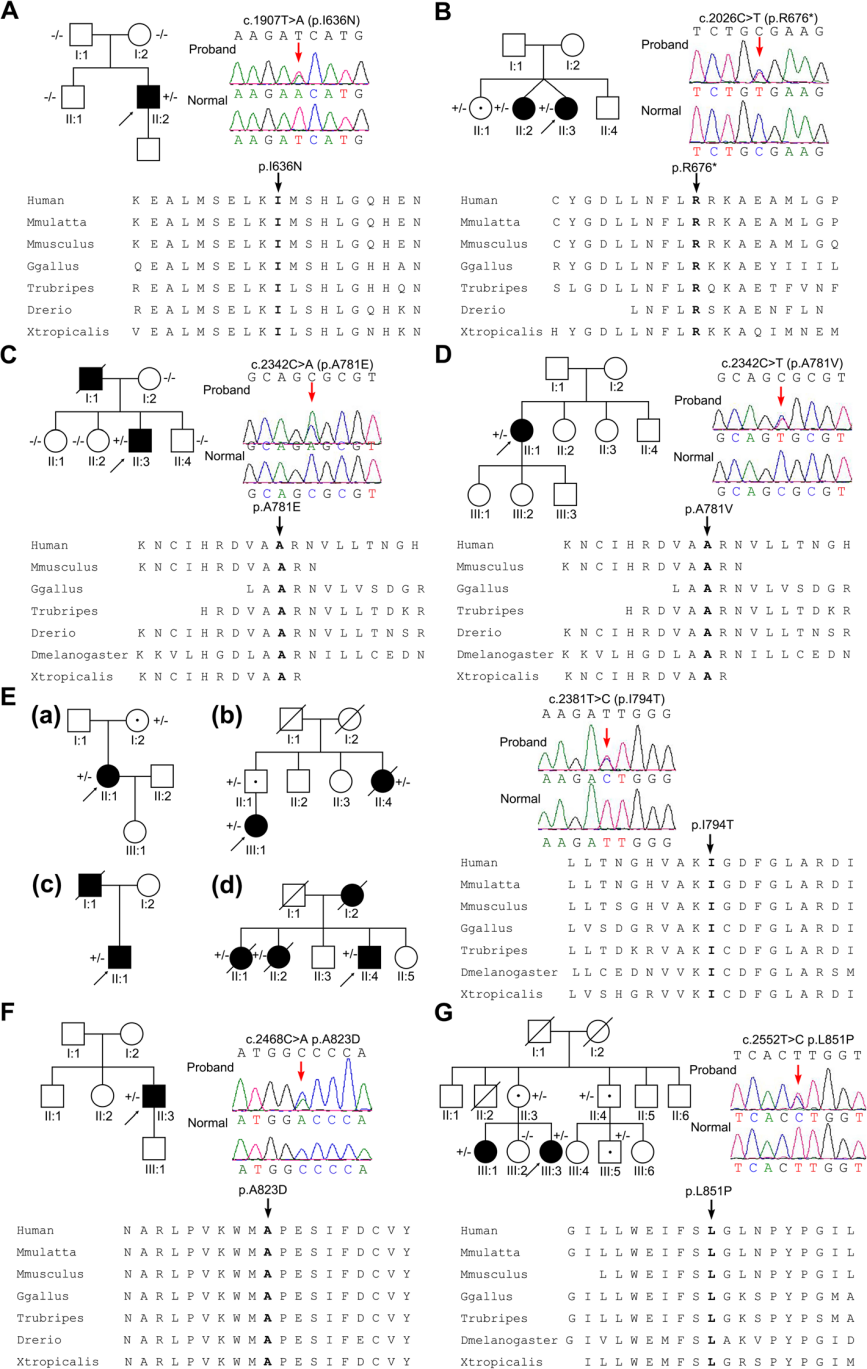
Ten family pedigrees with a diagnosis of HDLS. The pedigrees are shown in the top left, the corresponding chromatograms are shown in the top right, and the mutations located in the highly conserved region of protein are shown in the bottom. **a** *CSF1R* c.T1907A (p.I636N) identified only in the proband (II:2), but not in I:1, I:2 or II:1 of Family 1. **b** *CSF1R* c.C2026T (p.R676*) identified in two patients (II:2 and II:3), as well as one healthy carrier (II:1) of Family 2. **c** *CSF1R* c.C2342A (p.A781E) identified in two patients (II:2 and II:3), as well as one healthy carrier (II:1) of Family 3. **d** *CSF1R* c.C2342T (p.A781V) identified in the proband (II:1) of Family 4. **e** *CSF1R* c.T2381C (p.I794T) identified in four families: Family 5 (a), Family 6 (b), Family 7 (c), Family 8 (d). **f** *CSF1R* c.C2468A (p.A823D) identified in the proband (II:3) of Family 9. **g** *CSF1R* c.T2552C (p.L851P) identified in two patients (III:1 and III:3) of Family 10

A total of 7 different mutations of *CSF1R* gene were identified in 10 families (Fig. 1), including 4 novel mutations (c.1907 T > A p.I636N, c.2026C > T p.R676*, c.2468C > A p.A823D and c.2552 T > C p.L851P) and 3 documented mutations (c.2381 T > C p.I794T, c.2342C > A p.A781E, and c.2342C > T p.A781V). Among these, c.2381 T > C was found in four unrelated families (Fig. 1e). According to the prediction software, all these 7 mutations were predicted to be pathogenic with high probability (Additional file 2: Table S2). Of note, incomplete penetrance was observed in Family 2, 5, 6 and 10 (Fig. 1B, E-a, E-b, G).

### Defective autophosphorylation of CSF1R in cells expressing mutant CSF1R

**Fig. 2:**
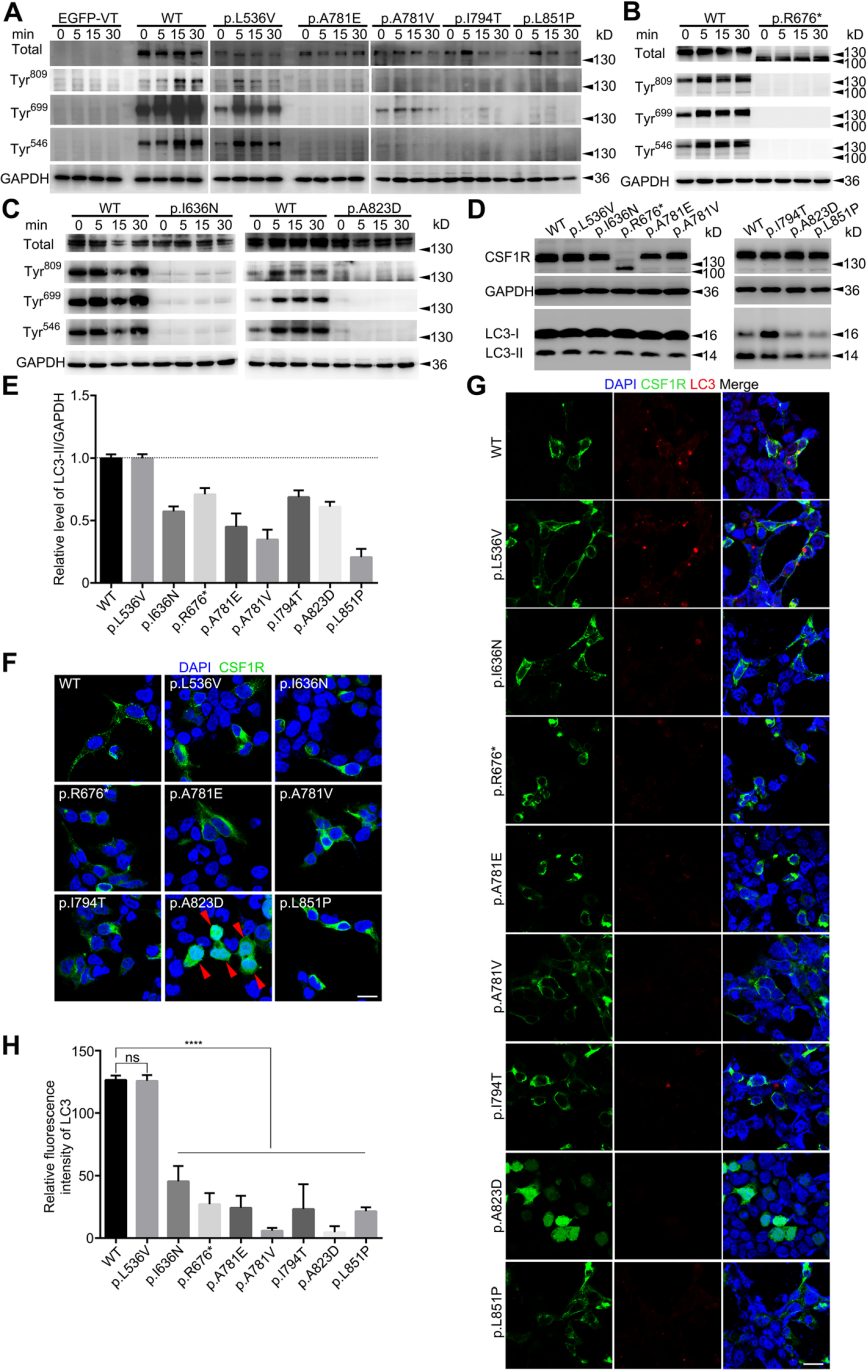
**a**-**c** Both wild-type and benign variant (p.L536 V) of CSF1R had strong phosphorylation after CSF1 treatment. In contrast, significantly weaker or none signal of CSF1R autophosphorylation at the selected tyrosine residues was detected after CSF1 treatment in CSF1R mutants (p.I636N, p.R676*, p.A781E, p.A781V, p.I794T, p.A823D and p.L851P) transfected cells. Experiments were repeated three times with similar outcomes. A band with relative smaller molecular weight (~ 110 kDa) was detected in p.R676*. **d** LC3-II/GAPDH levels of 7 mutant groups, WT and benign variant. **e** Bar plot indicated the statistical analysis (means±S) of 3 experiments. The average percentage of LC3-II/GAPDH of WT group was set as 1.0. One-way ANOVA with Dunnett’s test demonstrated that LC3-II/GAPDH levels in 7 mutants were significantly lower than WT (*P* < 0.0001), but there was no statistical difference between benign variation (p.L536 V) and WT. **f** Protein localization of CSF1R-WT/Mut in vitro. p.A823D tended to distribute diffusely in HEK 293 T cells (red arrows). The scale bar represents 20 μm. **g** Immunofluorescence analysis showed the aggregation of LC3-II was smaller in HEK 293 T cells overexpressed mutants than benign control and WT group. The scale bar represents 20 μm. **h** Bar plot indicated the statistical analysis (means±S) of 3 experiments. One-way ANOVA with Dunnett’s test; ns = non-significance; **** P < 0.0001

We transiently transfected wild-type or mutant CSF1R-EGFP cDNA into HEK 293 T cells. We also set a benign variant (c.1606C > G, p.L536 V) as a non-pathogenic control, which was found in our in-door exome sequencing data base of healthy controls. We next examined ligand-induced autophosphorylation of CSF1R by CSF-1 treatment (50 ng/mL) after removal of serum from the medium. Upon stimulation with CSF-1, we observed autophosphorylation of both wild-type CSF1R and benign variant (p.L536 V) at Tyr 546, 699 and 809 in a time-dependent manner, whereas all the 7 mutants showed moderate to severe autophosphorylation deficiency (Fig. 2a-c). Western blot analysis revealed comparable expression levels among wild-type, non-pathogenic variant (p.L536 V) and 6 mutants (p.I636N, p.A781E, p.A781V, p.I794T, p.A823D and p.L851P) (Fig. 2d). p.R676* is predicted to cause nonsense-mediated decay by generating a premature stop codon (http://www.mutationtaster.org). Western blotting also disclosed p.R676* with less molecular weight and significantly lower expression level than WT (Fig. 2d).

### Defective autophagy and protein distribution of CSF1R in vitro

Autophagy mediated by CSF-1/CSF1R plays a crucial role during differentiation of human monocytes into macrophages [14, 21], which induces typical autophagic structures, such as phagophores, autophagosomes and accumulation of LC3-II [14]. As the marker for autophagy activation, the level of LC3-II/GAPDH was tested in order to clarify whether these *CSF1R* mutations would interfere autophagy. We transiently transfected wild-type or mutant CSF1R cDNA into HEK 293 T cells. The result of western blotting shown in Fig. 2d-e, indicated that all of 7 mutants had lower level of LC3-II (percentage) than WT or benign control (p.L536 V). Then via immunofluorescence staining, we found obvious LC3 accumulation into mass or punctiform in cells expressing WT and benign control. Whereas, a less level of accumulation of LC3 was observed in 7 mutant groups (Fig. 2g-h). In vitro, p.A823D tended to diffusely distribute in the cytoplasm while all the others were located around the cell body and mostly on the cell membrane (Fig. 2f). Moreover, we also tested the mitochondria in vitro which was found to be abnormal in brain specimen both on morphology and quantity. The results showed that mitochondria tended to manifest disorganized and upregulated with *CSF1R* mutations expressed cells (data not shown). The fluorescence intensity per unit area of all 7 mutant groups was higher than wild type control, though without statistical difference during three repeated trials.

According to the functional study, co-separation analysis in family and the software prediction results, these 7 mutations were classified as pathogenic [20].

## Discussion

In this study, we identified 4 novel and 3 reported mutations in *CSF1R* among patients with leukoencephalopathy. All these 15 patients presented progressive neuropsychiatric and motor symptoms. Symptoms occurred in adulthood (24–46 years old) which is generally consistent with the previous studies (18–72 years old) [2, 9, 15, 19, 22]. Both predominance and earlier age of onset were observed in females than males. Similar results have been recorded in several clinical studies, showing the female to male ratio ranges from 1.3~2.5, and the female patients tends to have younger average age of onset (female^−^x: 27~42.8, male^−^x: 44.3~53.72) [2, 6, 17, 23]. Even patients from the same family due to the same causative mutation, female siblings could present initial symptoms with earlier age than their male siblings [6]. The clinical course from onset to death for the patients usually ranges 2–30 years [22]. Behavior and personality changes are common initial manifestations. The phenotype during course of disease is characterized by progressively aggravated frontal lobe syndrome, including decreased social interaction, poor judgment, depression, cognitive disability and personality changes [24]. The white matter biopsy for two patients, exhibited different severities of *CSF1R*-related primary axonopathy, including axonal spheroids, disorganized neurofilaments, myelin loss and cyto-organelle accumulation [9]. As the main differential diseases of *CSF1R*-related leukoencephalopathy, cerebral autosomal dominant arteriopathy with subcortical infarcts and leukoencephalopathy (CADASIL) and other leukoencephalopathies should be excluded with the help of pathological evidence and genetic detecting [19].

It is interesting that Patient 5 with c.2342C > T (p.A781V) also had peripheral neuropathy under electrophysiological examinations. To our knowledge, the peripheral nervous system is generally spared. However, in 2013 and 2016, Donato and Guerreiro also reported two patients with obvious peripheral neuropathy due to p.E847K and p.T567fsX44 respectively [23, 25]. Indeed, whether *CSF1R*-related spectrum includes peripheral neuropathy deserves longer follow-up with more patients enrolled as well as further study in animal models.

**Fig. 3:**
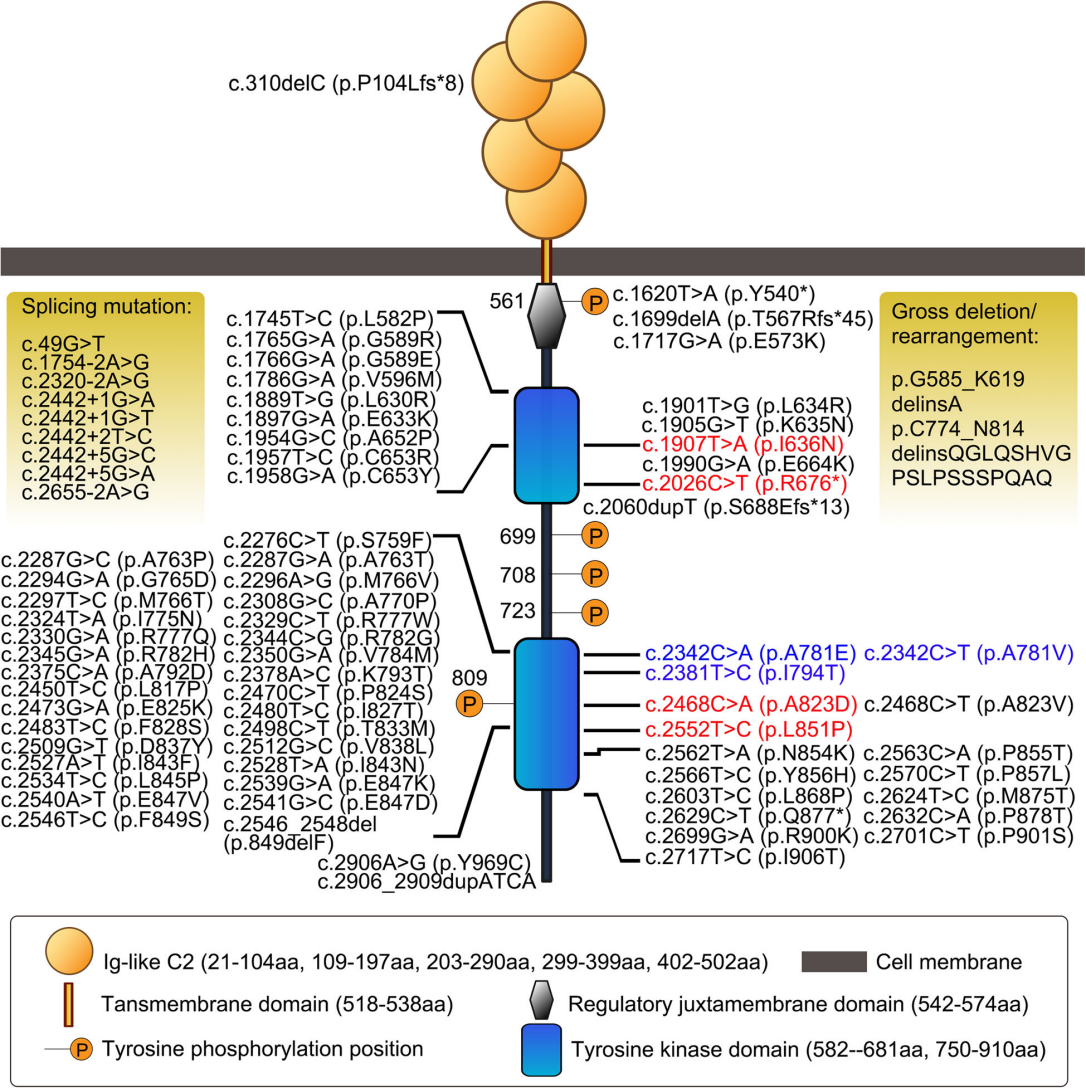
Diagram of CSF1R protein structure with reported mutations. Mutations in black: reported by other studies; mutations in red: first identified in this paper; mutations in blue: reported by both previous studies and this paper

Hitherto, several clinical investigations have confirmed that HDLS, POLD and ALSP have clinical and neuropathologic overlap as well as the same genetic basis [2, 16, 19]. *CSF1R* gene, one of the main causes of ALSP, encodes CSF1R protein which is a type III receptor tyrosine kinase belonging to the platelet-derived growth factor (PDGF) receptor family, which also includes PDGF-a and -b, the FMS-like tyrosine kinase 3 (FLT3) and the receptor for stem cell factor (c-KIT) [2]. CSF1R is mainly expressed on the surface of microglia in the CNS, which consists of five extracellular immunoglobulin (Ig) like domains, a single-pass transmembrane domain, a juxta membrane domain (JM), two intracellular tyrosine kinase domains (TKD1 and TKD2) and one insert domain [26]. As key regulators of most cellular pathways, protein kinase domains are structurally conserved and usually associated with disease [26, 27]. In order to figure out the relationship between mutation sites and protein domains, the mutations both documented before and in this paper were summarized along with CSF1R structure diagram (Fig. 3). Of note, 77.5% of these mutations (62/80) are located on the TKDs (exons 12–22), especially exon 18 and 19 [28]. The missense mutations account for 63.75% of all (51/80). There are also 9 splicing mutations, 5 small insertion/deletion mutations, 3 premature mutations and 2 large fragment rearrangements. Patients with *CSF1R*-related leukoencephalopathy have already been documented in Chinese Mainland [29–31]. However, most of the published literatures about the patients are limited to clinical case reports which are lack of comprehensive functional validation of identified mutations. In this work, 4 unrelated families from different regions of China harbored the same mutation c.2381 T > C (p.I794T). Interestingly, p.I794T was previously reported in 2 Chinese cases and at least five different clinical studies worldwide [2, 17, 29, 32, 33], reminiscent of a hot spot not only in China but also worldwide. In vitro, CSF-1-induced autophosphorylation of mutant CSF1R is injured, demonstrating loss of TK activity and signaling is associated with the pathogenesis [2, 9, 16, 17]. However, one mutation located in the extracellular Ig-like domain (p.P104Lfs*8) has also been identified [34, 35]. Interestingly, the transfected p.A823D mutant was distributed diffusely in the cytoplasm, indicating cell surface expression of mutant CSF1R might be decreased. According to the silico analysis (http://www.mutationtaster.org), the substitution of alanine by aspartate was predicted to lose the cytoplasmic topological domain (539-972aa). In 2013, Hiyoshi et al. also investigated several *CSF1R* mutants with weak cell surface expression [36]. The precise mechanism by which causative mutations perturb CSF1R trafficking to the cell surface remains unknown. At least, this was not simply due to severely impaired kinase activity [36].

Up to now, no apparent phenotype-genotype correlation has been found [9]. In the functional exploration part, we showed the autophosphorylations of all 7 mutants were lost except that c.2342C > T had partial impairment of autophosphorylation. In 2018, Konno et al. also reported one case with white matter abnormalities and partial loss of autophosphorylation of *CSF1R* c.1717G > A (p.E573K) [15]. In addition, the premature stop mutation (c.2026C > T, p.R676*) encoded a truncated protein form lacking both TKD 2 and a part of TKD 1, supporting the loss-of-function and haploinsufficiency pathogenesis hypothesis [2, 7, 9, 17].

Recently, CSF-1/CSF1R pathway has been confirmed to play an essential role in autophagy pathways in mono-macrophages system [14, 21]. In eukaryotic cells, autophagy allows degradation of macromolecules (proteins, lipids, nucleotides) and elimination of superfluous or damaged organelles (mitochondria, peroxisomes and endoplasmic reticulum) [14]. Cells often use this process to promote cell survival in adverse conditions such as nutrient deprivation or extracellular stimuli [14]. Thus, an important question arising concerns if the CSF1R mutations identified in the patients would influence the autophagy progress. Since LC3 is the most widely monitored autophagy-related protein, and LC3-II is more stable and more closely related to autophagosomes than LC3-I, the consensus on autophagy detection is that levels of LC3-II should be compared to internal parameter (such as actin or GAPDH), but not to LC3-I [37]. In this study, both western blotting and immunofluorescence results showed the level of LC3-II in cells was lower in cells overexpressed CSF1R-Mut than the benign control or CSF1R-WT when exposed to CSF-1 stimulation. Though CSF1R-Mut could induce autophagy like CSF1R-WT, less level of LC3-II accumulation was observed, indicating the autophagy process might be disturbed by abnormal CSF-1/CSF1R signaling. In the CNS, microglial inflammation and autophagy dysfunction are closely related to varieties of neurodegenerative diseases, such as Parkinson’s disease and Alzheimer disease [38, 39]. Microglia are crucial in regulating homeostasis, because they critically determine the fate of other neural cells upon endogenous or external stimuli triggering microenvironment [39]. Meanwhile, CSF1R haploinsufficiency has been confirmed to cause reduced microglia density and widespread depletion in the brain of both zebrafish and human [13]. Thus, CSF1R-related autophagy dysfunction and microglia loss may be early or initial pathogenic events contributing to leukodystrophy.

## Conclusions

This study focused on Chinese patients with *CSF1R*-related leukoencephalopathy through detailed clinical, radiological, pathological and functional investigations. Our findings support the hypotheses that haploinsufficiency is sufficient to cause the disorder. Whether peripheral neuropathy belongs to *CSF1R*-related spectrum is still pending and needs further investigation with more patients enrolled and longer follow-up. We provide initial insight into *CSF1R* mutations leading to deficiency of autophagy. Therefore, understanding how to promote the mutant CSF1R phosphorylation may reveal new opportunities for targeted therapies and further investigations about the role of autophagy may shed light on the pathogenetic studies of *CSF1R*-related leukoencephalopathy.

## Additional files

Additional file 1: **Figure S1.** A-C. Sagittal and transverse view of neuroimaging for patients with *CSF1R*-related leukoencephalopathy. (A = Patient 5, B = Patient 7, C-a&b = Patient 15, C-c = Patient 14). Brain MRI showed varying degree of white matter lesions, corpus callosum atrophy (A-B, C-a&c). Intracranial calcification was identified for Patient 15 on brain CT (C-b, red arrow). The ventricular enlargement of Patient 14, under lateral ventricle drainage (C-c). D-I. Immunohistochemistry of brain biopsy of Patient 5 by HE staining (D), CD68 (E), anti-phosphorylated neurofilament (F), CD3 (G), CD20 (H) and Olig-2 (I) immunohistochemistry. Axonal spheroids by HE staining (red arrows) or positively marked by anti-phosphorylated neurofilament (yellow arrows). J-O. Immunohistochemistry of brain biopsy of Patient 7 by HE staining (J), CD68 (K), anti-phosphorylated neurofilament (L), CD3 (M), CD20 (N) and Olig-2 (O) immunohistochemistry. Axonal spheroids by HE staining (red arrows) or positively marked by anti-phosphorylated neurofilament (yellow arrows). P. Myelin loss in frontal white matter with abundant disorganized neurofilaments and cyto-organelles of Patient 5 (red arrows, mitochondrial vacuolation). Q-R. Giant and ballooned axons in white matter with increased cyto-organelles (mitochondria and vesicae) of Patient 7. (ZIP 28010 kb)

Additional file 2 **Table S1.** Electrophysiological examination of Patient 5. **Table S2.** Prediction results of CSF1R mutations in silico analysis. (DOCX 27 kb)

## Data Availability

The dataset used and/or analyzed during the current study are available from the corresponding author on reasonable request.

## Abbreviations

AARS2: Alanyl-tRNA Synthetase 2
ALSP: Adult-onset Leukodystrophy with Neuroaxonal Spheroids and Pigmented Glia
CADASIL: Cerebral Autosomal Dominant Arteriopathy with Subcortical Infarcts and Leukoencephalopathy
CMAP: Compound Motor Action Potential
CNS: Central Nervous System
CSF1R: Colony-stimulating Factor 1 Receptor
DWI: Diffusion Weighted Imaging
EMG: Electromyography
FLT3: FMS-like Tyrosine Kinase 3
HDLS: Hereditary Diffuse Leukoencephalopathy with Spheroids
Ig: Immunoglobulin
JM: Juxta Membrane Domain
LC3: Microtubule Associated Protein 1 Light Chain 3
PDGF: Platelet-derived Growth Factor
POLD: Pigmented Orthochromatic Leukodystrophy
SNAP: Sensory Nerve Action Potential
TKD: Tyrosine Kinase Domains
