# Supplementary figures and images for: Clinicopathologic characterization and abnormal autophagy of CSF1R-related leukoencephalopathy
Source: Transl Neurodegener. 2019 Dec 2;8:32. doi: 10.1186/s40035-019-0171-y (PMC6886209; doi:10.1186/s40035-019-0171-y)

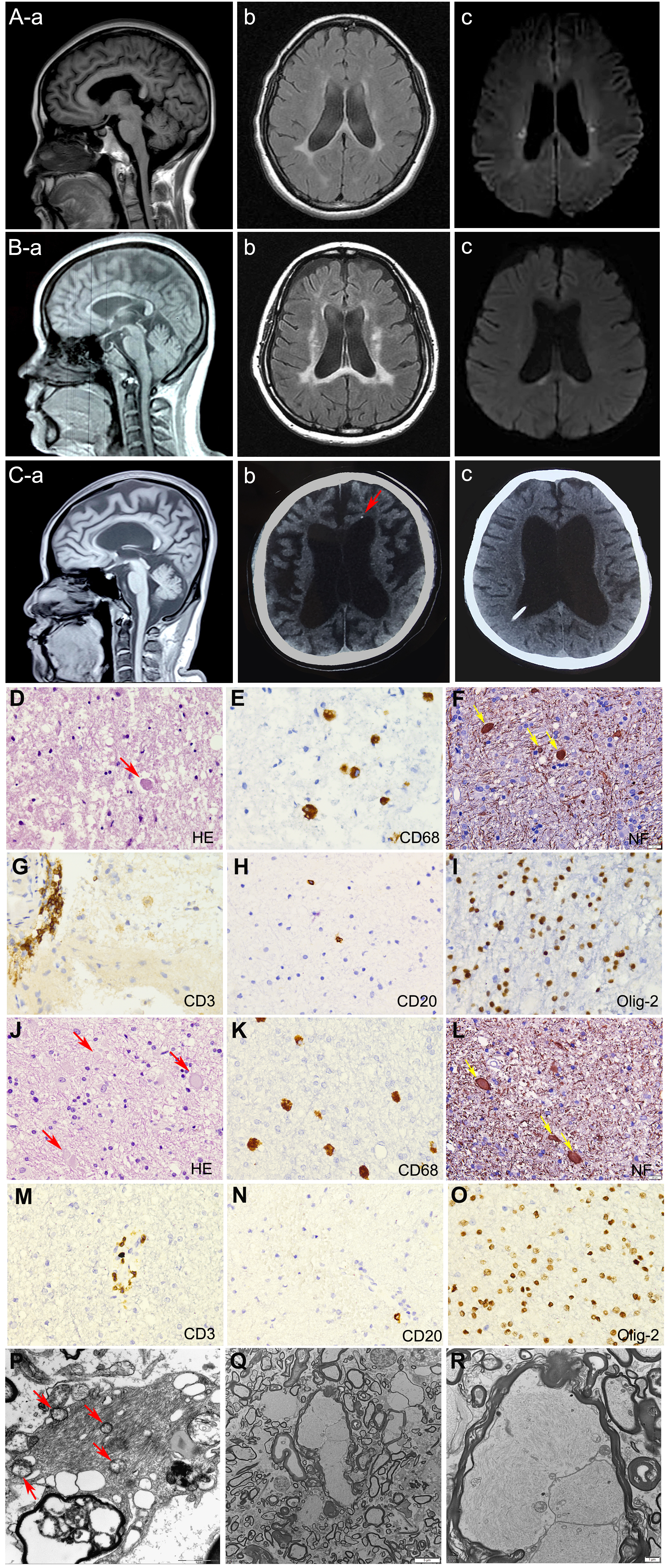

Supplement: Supplementary file 1 — Figure S1. A-C. Sagittal and transverse view of neuroimaging for patients with CSF1R-related leukoencephalopathy. (A = Patient 5, B = Patient 7, C-a&b = Patient 15, C-c = Patient 14). Brain MRI showed varying degree of white matter lesions, corpus callosum atrophy (A-B, C-a&c). Intracranial calcification was identified for Patient 15 on brain CT (C-b, red arrow). The ventricular enlargement of Patient 14, under lateral ventricle drainage (C-c). D-I. Immunohistochemistry of brain biopsy of Patient 5 by HE staining (D), CD68 (E), anti-phosphorylated neurofilament (F), CD3 (G), CD20 (H) and Olig-2 (I) immunohistochemistry. Axonal spheroids by HE staining (red arrows) or positively marked by anti-phosphorylated neurofilament (yellow arrows). J-O. Immunohistochemistry of brain biopsy of Patient 7 by HE staining (J), CD68 (K), anti-phosphorylated neurofilament (L), CD3 (M), CD20 (N) and Olig-2 (O) immunohistochemistry. Axonal spheroids by HE staining (red arrows) or positively marked by anti-phosphorylated neurofilament (yellow arrows). P. Myelin loss in frontal white matter with abundant disorganized neurofilaments and cyto-organelles of Patient 5 (red arrows, mitochondrial vacuolation). Q-R. Giant and ballooned axons in white matter with increased cyto-organelles (mitochondria and vesicae) of Patient 7. (ZIP 28010 kb) [file 40035_2019_171_MOESM1_ESM.zip › Supplementary Fig 1.tif]
