# Supplementary material for: Clinicopathologic characterization and abnormal autophagy of CSF1R-related leukoencephalopathy
Source: Transl Neurodegener. 2019 Dec 2;8:32. doi: 10.1186/s40035-019-0171-y (PMC6886209; doi:10.1186/s40035-019-0171-y)
Supplement: Supplementary file 2 — Table S1. Electrophysiological examination of Patient 5. Table S2. Prediction results of CSF1R mutations in silico analysis. (DOCX 27 kb) [file 40035_2019_171_MOESM2_ESM.docx]

**Additional file 2**

**Table S1 Electrophysiological examination of Patient 5.**

| **Nerve/Sites** | **Rec. Site** | **Lat**  ms | **Amp**  mV | **Rel Amp**  % | **Dur.**  ms | **Dist**  cm | **Vel**  m/s |
| --- | --- | --- | --- | --- | --- | --- | --- |
| **L Median nerve** | | | | | | | |
| **1. Wrist** | APB | 3.15 | 8.6 | 100 | 4.90 |  |  |
| **3. Elbow** | APB | 7.65 | 8.5 | 98.4 | 4.90 | 20.8 | 46.2 |
| **R Median nerve** | | | | | | | |
| **1. Wrist** | APB | 3.30 | 8.1 | 100 | 4.45 |  |  |
| **3. Elbow** | APB | 7.75 | 7.7 | 95.6 | 4.65 | 20.7 | 46.5 |
| **L Ulnar nerve** | | | | | | | |
| **1. Wrist** | ADM | 2.70 | 8.8 | 100 | 5.15 |  |  |
| **3. Elbow** | ADM | 6.55 | 8.1 | 92.2 | 5.10 | 18.6 | 48.3 |
| **R Ulnar nerve** | | | | | | | |
| **1. Wrist** | ADM | 2.80 | 9.1 | 100 | 4.90 |  |  |
| **3. Elbow** | ADM | 6.60 | 8.7 | 95.7 | 4.85 | 19.3 | 50.8 |

| **Nerve/Sites** | **Rec. Site** | **Onset**  ms | **Peak**  ms | **NP Amp**  μV | **PP Amp**  μV | **Dist**  cm | **Vel**  m/s |
| --- | --- | --- | --- | --- | --- | --- | --- |
| **L Median nerve** | | | | | | | |
| **2. Wrist** | Dig II | 2.10 | 2.70 | 11.5 | 16.1 | 11 | 52.4 |
| **4. Elbow** | Dig II | 5.85 | 6.60 | 2.9 | 0.04 | 22.1 | 58.9 |
| **R Median nerve** | | | | | | | |
| **2. Wrist** | Dig II | 2.05 | 2.60 | 7.8 | 16.8 | 10.4 | 50.7 |
| **4. Elbow** | Dig II | 5.90 | 6.95 | 0.00 | 4.2 | 20.7 | 53.8 |
| **L Ulnar nerve** | | | | | | | |
| **2. Wrist** | Dig V | 2.55 | 3.30 | 4.6 | 14.8 | 13.4 | 52.5 |
| **4. Elbow** | Dig V | 5.80 | 6.70 | 3.0 | 4.2 | 18.6 | 57.2 |
| **R Ulnar nerve** | | | | | | | |
| **2. Wrist** | Dig V | 2.55 | 3.35 | 9.8 | 14.1 | 12.5 | 49.0 |
| **4. Elbow** | Dig V | 5.80 | 6.50 | 2.1 | 2.5 | 19.3 | 59.4 |

**SCV**

| **Nerve/Sites** | **Rec. Site** | **Latency**  **ms** | **Peak Ampl**  **μV** | **Distance**  **cm** | **Velocity**  **m/s** |
| --- | --- | --- | --- | --- | --- |
| **L Superficial peroneal nerve** | | | | | |
| **1. Lateral Leg** | Foot | 2.75 | 6.7 | 10.3 | 37.5 |
| **R Superficial peroneal nerve** | | | | | |
| **1. Lateral Leg** | Foot | 2.85 | 3.6 | 10.6 | 37.2 |

**MCV**

| **Nerve/Sites** | **Latency**  **ms** | **Ampl**  **mV** | **Distance**  **cm** | **Velocity**  **m/s** |
| --- | --- | --- | --- | --- |
| **R Nervus peroneus communis-EDB** | | | | |
| **1. Median of ankle** | 4.50 | 2.2 |  |  |
| **2. Below fibula capitulum** | 12.00 | 2.3 | 27.3 | 36.4 |
| **L Nervus peroneus communis-EDB** | | | | |
| **1. Median of ankle** | 4.35 | 1.5 |  |  |
| **2. Below fibula capitulum** | 11.65 | 1.4 | 25.5 | 34.9 |
| **R Tibial nerve-AH** | | | | |
| **1. Ankle** | 7.05 | 8.0 |  |  |
| **2. Popliteal** | 15.40 | 6.9 | 30.2 | 36.2 |
| **L Tibial nerve-AH** | | | | |
| **1. Ankle** | 5.50 | 8.8 |  |  |
| **2. Popliteal** | 14.10 | 6.4 | 31 | 36.0 |

**F Wave**

| **Nerve** | **Min M Lat ms** | **Max M Lat ms** | **Mean M Lat ms** | **Min**  **F-M**  **ms** | **Max**  **F-M**  **ms** | **Mean F-M**  **ms** | **Min F/M**  **mV** | **Max F/M**  **mV** | **Mean F/M**  **mV** | **% F**  **%** |
| --- | --- | --- | --- | --- | --- | --- | --- | --- | --- | --- |
| **R Nervus peroneus communis-EDB** | 4.90 | 5.00 | 4.96 | 45.30 | 51.60 | 47.30 | 0 | 0.05 | 0.03 | 100 |
| **R Tibial nerve-AH** | 5.35 | 5.55 | 5.47 | 41.35 | 49.25 | 44.98 | 0.01 | 0.04 | 0.02 | 100 |
| **L Tibial nerve-AH** | 5.95 | 6.45 | 6.23 | 44.35 | 46.85 | 45.18 | 0.02 | 0.05 | 0.03 | 100 |
| **L Nervus peroneus communis-EDB** | 4.80 | 4.90 | 4.85 | 43.05 | 50.00 | 47.32 | 0.05 | 0.06 | 0.05 | 100 |
| **L Ulnar nerve-ADM** | 3.35 | 3.50 | 3.43 | 23.15 | 25.25 | 23.91 | 0.01 | 0.04 | 0.02 | 100 |
| **R Ulnar nerve-ADM** | 2.65 | 2.80 | 2.71 | 22.35 | 25.90 | 24.05 | 0.01 | 0.04 | 0.03 | 100 |

**Table S2 Prediction results of in silico analysis.**

| **Mutation** | **Polyphen-2** | | **SIFT** | | **Mutationtaster** | |
| --- | --- | --- | --- | --- | --- | --- |
|  | **Prediction** | **Score** | **Prediction** | **Score** | **Prediction** | **Probability** |
| c.1907T>A (p.I636N) | Probably damaging | 1 | Damaging | 0.000 | Disease causing | 0.99999 |
| c.2026C>T (p.R676*) | / | / | / | / | Disease causing | 1 |
| c.2342C>A (p.A781E) | Probably damaging | 1 | Damaging | 0.000 | Disease causing | 0.99999 |
| c.2342C>T (p.A781V) | Probably damaging | 1 | Damaging | 0.000 | Disease causing | 0.99999 |
| c.2381T>C (p.I794T) | Probably damaging | 1 | Damaging | 0.000 | Disease causing | 0.99999 |
| c.2468C>A (p.A823D) | Probably damaging | 1 | Damaging | 0.001 | Disease causing | 0.99999 |
| c.2552T>C (p.L851P) | Probably damaging | 1 | Damaging | 0.000 | Disease causing | 0.99999 |
